# Evaluation of Lymphedema Prevention Protocol on Quality of Life among Breast Cancer Patients with Mastectomy

**DOI:** 10.31557/APJCP.2019.20.10.3077

**Published:** 2019

**Authors:** Dr S Punitha Josephine

**Affiliations:** *Department of Medical Surgical Nursing, Karpaga Vinayaga College of Nursing, Kancheepuram-Dt.The Tamilnadu Dr.M.G.R. Medical University,Guindy, Chennai, India. *

**Keywords:** Breast cancer, mastectomy, lymphedema prevention protocol, lymphedema occurrence, quality of life

## Abstract

**Objective:**

Lymphedema is a widespread complication after surgery or radiation therapy due to the damage and obstruction of the lymphatic vessels. A study was conducted to assess the effectiveness of lymphedema prevention protocol on quality of life among breast cancer patients with mastectomy at a selected hospital in Tamil Nadu. Objectives of the study were to identify the effect of lymphedema prevention protocol on lymphedema occurrence and quality of life.

**Methods:**

A quantitative research approach of quasi experimental non-equivalent with control group before –after design (Non randomized) was used. The investigator had included 120 participants by using purposive sampling technique which included 60 each in study and comparison group. Pre test was done before the intervention of lymphedema prevention protocol to both comparison and study group participants. Lymphedema prevention protocol was implemented for study group whereas comparison group received routine care. Post tests 1, 2, 3, and 4 were done by using the structured questionnaire at 10^th^ day, 30^th^, 60^th^ and 90^th^ post operative day respectively. Subjects’ responses were coded and statistically analyzed by using descriptive and inferential statistics.

**Results:**

The comparison of quality of life between study and comparison group over a period of time were statistically significant at p< 0.001 whereas lymphedema occurrence was significant at p< 0.01.

**Conclusion:**

The early execution of preventive measures of lymphedema prevents the lymphedema occurrence and promotes the quality of life among patients undergone mastectomy.

## Introduction

Health sector is being continuously challenged with the changes in life style of human beings. A large number of life style related factors are identified as risk factors for many diseases, most commonly the cancer. It is emerging as a major public health problem worldwide. The common risk factors are stress, tobacco, dietary habits, inadequate physical activity, and alcohol consumption. India is rapidly stepping towards industrialization vis-à-vis urbanization resulting in change of lifestyle factors. With the control of infectious diseases and increased longevity of the growing population in India the spectrum of disease has changed and the burden of non-communicable diseases like cancer is on the rise.

Breast cancer is the common malignancy in woman. The incidence of breast cancer is increasing globally. Each year more than 1 million people are diagnosed with cancer in United States and it is expected to double by the year 2050-American cancer society (society, 2014). In 2018 march, 2, 08,8 849 (11.6%) women were diagnosed with breast cancer contributing about 11.6% of the total cancer incidence burden worldwide and 5% of them were alive who had been diagnosed with breast cancer in the previous five years-International agency for research on cancer (International agency for research on cancer, 2019 ). Breast cancer is one of the most common cancers among Indian women - ICMR Indian Council for Medical Research reports 1.5 lakh new breast cancer cases in India, of which 70,000 succumb every year (ICMR, 2019). The incidence of breast cancer in India is on the rise.

Despite advances in treatment, many breast cancer survivors experience devastating side effects and complications due to the treatment modality. Surgery is important in the diagnosis and treatment of most breast cancer. Lumpectomy, modified radical mastectomy, axillary node dissection-level I, II, III, and simple or radical mastectomy are the most frequently used surgical procedures for treatment of breast cancer. But it causes complications such as lymphedema, infection, seroma, hematoma and cellulitis.

Lymphedema is a swelling of arm usually occurs on the side of mastectomy. Physically lymphedema causes distressing symptoms such as swelling, firmness, tightness, numbness and impaired limb mobility (Armer, 2007). The upper arm lymphedema, makes simple tasks such as picking up children, getting dressed or exercising painful, has a detrimental impact on the patient’s quality of life (Mc. Dowel and Mattie, 2008). Lymphedema causes physical discomfort and disability, as well as a cosmetic deformity which can lead to anxiety, depression and emotional distress. These can affect a woman’s activities of daily living and quality of life. Studies have shown a range of problems associated with lymphedema including pain, discomfort, difficulties with clothing, reduced function and mobility, social isolation and employment difficulties (Todd, 2009).

Early detection methods and multimodality treatment strategies have increased the survival rate of breast cancer patients. The health-related quality of life is a vital issue among breast cancer patients since this population is growing rapidly. Lymphedema is the most dreaded sequelae of breast cancer treatment. The physical and psychologic problems associated with lymphedema can significantly affect the quality of life. A study disclosed that lymphedema was an independent predictor of decreased quality of life among patients with breast cancer (Beaulac et al., 2002). As a chronic, incurable condition that does not affect life expectancy; lymphedema exacts a staggering medical, social, psychological and functional toll. The cumulative cost to patients’ time, finances and energy is extremely high.

Therefore, nurses are playing a major role to execute the preventive measures of arm lymphedema systematically among patients subjected to mastectomy at the earliest before initiation and during treatment and follow –up visits. It is imperative to preserve the arm function, health related quality of life and maintenance of independence for as long and as comfortable as possible which in turn reduces the health care expenditure.

## Materials and Methods

A quantitative research approach of Quasi experimental-before–after design with a comparison group (Non-randomized) was used to find out the effectiveness of lymphedema prevention protocol on quality of life among breast cancer patients undergoing mastectomy at a selected hospital in Tamil Nadu. It involved an execution of lymphedema prevention protocol for study group whereas comparison group received routine care. Routine care included few range of motion exercises for the arm, without any structured content. The objectives of the study were to identify the effect of lymphedema prevention protocol on lymphedema occurrence and quality of life among breast cancer patients undergoing mastectomy. 

Lymphedema prevention protocol refers to the nursing interventions designed on various aspects of lymphedema prevention such as post mastectomy exercises,arm compression sleeve, self lymphatic drainage and arm care guidelines among breast cancer patients undergoing mastectomy. The patients were given planned teaching about the prevention of lymphedema using lecture cum demonstration method with power point slides and handouts during pre operative period and reinforcements are given using handouts in post operative and maintenance phase.

Institutional Ethical committee approval was obtained from the setting of data of collection.

*Hypotheses*

H_1_- There is a significant difference in lymphedema occurrence among patients undergone mastectomy and received lymphedema prevention protocol than those who do not at p<0.05.

H_2_ There is a significant difference in quality of life among patients undergone mastectomy and received lymphedema prevention protocol than those who do not at p<0.05.

*Criteria for sample selection*

*Inclusion criteria*

Female patients diagnosed to have primary breast cancer stage II or III undergoing modified/radical unilateral mastectomy, aged 30-60 years and able to read and understand Tamil and or English were included for the study.

*Exclusion criteria*

Female patients diagnosed to have breast cancer stage I and advanced breast cancer with metastasis, known lymphatic disease/ peripheral vascular disease and with the history of shoulder injury and experiences limitation of shoulder or hand movement were excluded.

**Figure 1:**
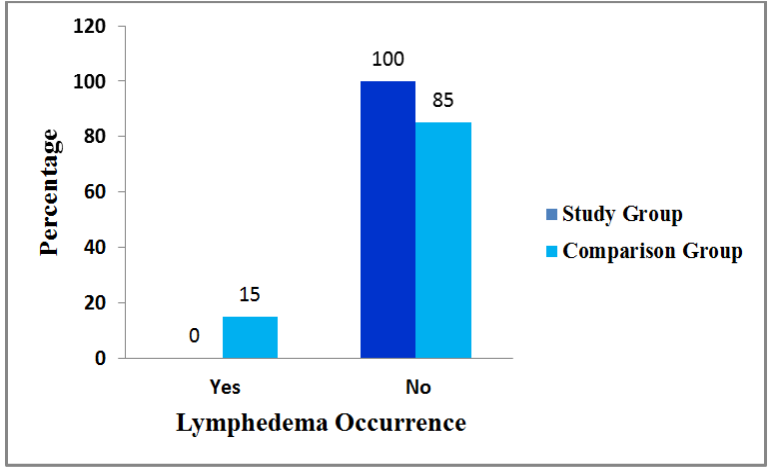
Distribution of Lymphedema Occurrence among Study and Comparison Group in Post Test 4

**Table 1:** Distribution of Demographic Variables among Study and Comparison Group in Pretest (N=120)

S. No.	Demographic variables	Study group n=60		Comparison group n=60		Univariate analysis& ‘p’ value
		No.	%	No.	%	
1	Age (years)					
	a. 30 - 40	9	15.0	4	6.7	p=0.439 NS
	b. 41 - 50	36	60.0	40	66.7	
	c. 51 - 60	15	25.0	16	26.7	
2	Marital Status					
	a. Married	46	76.7	54	90.0	p=0.007**
	b. Divorce	8	13.3	3	5.0	
	c. Widow	6	10.0	3	5.0	
3	Religion					
	a. Hindu	30	50.0	37	61.7	p=0.809 NS
	b. Christian	13	21.7	11	18.3	
	c. Muslim	17	28.3	12	20.0	
4	Residence					
	a. Rural	8	13.3	13	21.7	p=0.307 NS
	b. Urban	52	86.7	47	78.3	
5	Educational Status					
	a. No formal education	3	5.0	11	18.3	p=0.196 NS
	b. Primary education	2	3.3	5	8.3	
	c. High school	5	8.3	2	3.3	
	d. Higher secondary	10	16.7	8	13.3	
	e. Graduate & above	40	66.7	34	56.7	
6	Occupation					
	a. Unemployed	12	20.0	15	25.0	p=0.851 NS
	b. Labour	3	5.0	0	0	
	c. Government employee	7	11.7	5	8.3	
	d. Private employee	34	56.7	29	48.3	
	e. self employed	4	6.7	11	18.3	
7	Income / month (Rs.)					
	a. ≤ 5000	4	6.7	4	6.7	p=0.692 NS
	b. 5001 – 15000	11	18.3	12	20.0	
	c. 15001 – 25000	21	35.0	22	36.7	
	d. > 25000	24	40.0	22	36.7	
8	Type of family					
	a. Joint family	21	35.0	14	23.3	p=0.001***
	b. Nuclear family	36	60.0	37	61.7	
	c. Extended family	3	5.0	9	15.0	
9	Size of family					
	a. 2-5 members	40	66.7	46	76.7	p=0.829 NS
	b. 6-8 members	20	33.3	14	23.3	
10	Food habits					
	a. Vegetarian	1	1.7	2	3.3	0.035 df=1 p=0.851 NS
	b. Non-vegetarian	59	98.3	58	96.7	

NS-Not significant; ** p<0.0; *** p<0.001

**Table 2:** Distribution of Clinical Variables among Study and Comparison Group in Pretest (N = 120)

S. No.	Clinical variables	Study group n=60		Comparison group n=60		Univariate analysis&‘p’ value
		No.	%	No.	%	
1	Diagnosis					
	a.Ca. breast (Right)	36	60.0	36	60.0	p =0.162 NS
	b. Ca. breast (Left)	24	40.0	24	40.0	
2	Stage of cancer breast					
	a. Stage IIA	8	13.3	13	21.7	p =0.437 NS
	b. Stage IIB	37	61.7	32	53.3	
	c. Stage IIIA	14	23.3	13	21.7	
	d. Stage IIIB	1	1.7	2	3.3	
3	Type of surgery					
	a. Modified radical mastectomy	59	98.3	57	95.0	p =0.817 NS
	b. Radical mastectomy (Total)	1	1.7	3	5.0	
4	Axillary node dissection status					
	a. Level I – III	60	100	56	93.3	p =0.701 NS
	b. Level I – II	-	-	4	6.7	
5	Side of surgery					
	a. Dominant	36	60.0	36	60.0	p =0.162 NS
	b. Non Dominant	24	40.0	24	40.0	
6	Co-morbid illness					
	a. Diabetes mellitus	10	16.7	16	26.7	p =0.833 NS
	b. Hypertension	4	6.7	11	18.3	
	c. Hypertension and Diabetes	10	16.7	11	18.3	
	d. None	36	60.0	22	36.7	

NS-Not significant; *p<0.05

**Table 3:** Distribution of Biological Variables among Study Group in Pretest N=120

S. No.	Biological variables	Study group n=60		Comparison group n=60		Univariate analysis & ‘p’ value
		No.	%	No.	%	
1.	Weight (Kgs.)					
	a. 65-70	11	18.3	12	20.0	p=0.401 NS
	b. 70.1-75	19	31.7	18	30.0	
	c. 75.1-80	24	40.0	21	35.0	
	d. 80.1-85	6	10.0	9	15.0	
2.	Body mass index					
	c. 25–29.9	27	45.0	29	48.3	p =0.622 NS
	d. BMI of 30 or above	33	55.0	31	51.7	
3.	Haemoglobin (gms%)					
	a. 8-10	20	33.3	20	33.3	p =0.820 NS
	b. 10.1- 12	36	60.0	38	63.3	
	c. 12.1-14	4	6.7	2	3.3	
4.	RBC (million cells/mm^3^)					
	a. 3.6-4.5	51	85.0	38	63.3	p =0.006**
	b. 4.6- 5.5	9	15.0	22	36.7	
5.	WBC – Total count (cells/mm^3^)					
	a. 8001-10000	5	8.3	7	11.7	p =0.712 NS
	b. 10001-11000	15	25.0	23	38.3	
	c. >11000	40	66.7	30	50.0	
6.	Lymphocytes (%)					
	a. 25.1-30	1	1.7	-	-	p =0.560 NS
	b. 30.1-35	39	65.0	31	51.7	
	c. 35.1-40	20	33.3	29	48.3	
7.	Neutrophils (%)					
	a. 55.1-60	1	1.7	-	-	p =0.004**
	b. 60.1-65	55	91.6	43	71.7	
	c. 65.1-70	4	6.7	17	28.3	
8	Total protein (gms/dl.)					
	a. 5.1-6	9	15.0	8	13.3	p =0.939 NS
	b. 6.1-7	45	75.0	46	76.7	
	c.7.1-8	6	10.0	6	10.0	
9	Serum albumin (gms/dl)					
	a. 3.6-4.5	48	80.0	50	83.3	p =1.000 NS
	b. 4.6-5.5	12	20.0	10	16.7	

NS-Not significant; ** p<0.01; ss-statistically significant

**Table 4:** Repeated Measures of ANOVA on Lymphedema Occurrence between Study and Comparison Group Over a Period of Time (N=120)

S. No.	Observation	Study group n=60		Comparison group n=60		‘p’ value
		Mean	SD	Mean	SD	
1	Pre test	2.00	0.00	2.00	0.00	P=0.002** ss
2	Post test 1	2.00	0.00	2.00	0.00	
3	Post test 2	2.00	0.00	2.00	0.00	
4	Post test 3	2.00	0.00	2.00	0.00	
5	Post test 4	2.00	0.00	1.85	0.36	

**p<0.01; ss-statistically significant

**Table 5:** Repeated Measures of ANOVA on Quality of Life between Study and Comparison Group over a Period of Time (N =120)

S. No.	Observation	Study group n=60		Comparison group n=60		‘ p’ Value
		Mean	SD	Mean	SD	
I	Total score					
1	Pre test	96.62	3.12	95.75	3.36	P=0.000*** SS
2	Post test 1	80.27	2.82	73.76	3.16	
3	Post test 2	84.44	5.32	75.32	5.81	
4	Post test 3	89.57	5.15	80.27	7.18	
5	Post test 4	96.82	4.23	87.84	3.95	

*** p<0.001; ss-statistically significant

The study was conducted at a selected Hospital at Chennai and Ethical committee permission was obtained by the investigator from the Institutional Ethical committee. The investigator recruited 60 participants each for study and comparison group using non-probability purposive sampling technique based on the inclusion criteria.

A structured instrument was used to collect the data as discussed below.

*Part A: Demographic Variables*

The tool comprised of age, marital status, religion, residence, educational Status, occupation, income per month, type of family, size of family and food habits of breast cancer patients which were obtained by structured interview schedule.

*Part B: Clinical variables*

It consisted of diagnosis, stage of cancer Breast, type of surgery, axillary node dissection status, side of surgery and Co-Morbid illness obtained through clinical records survey.

*Part C: Biological variables*

It included weight, BMI, Haemoglobin value (gms%), RBC (Million cells /mm^3^, WBC – Total Count (cells/mm^3^), Differential Count – Lymphocytes (%), neutrophils (%), Total Protein (gms/dl) and Serum Albumin (gms/dl) and those were obtained from clinical records.

*Part D: Lymphedema Assessment Tool*

It is a standardized tool developed by Chen YW et.al 2008. It encompassed measurement of arm circumference at pre-operative period and; 10^th^, 30^th^, 60^th^ and 90^th^ post-operative day to identify the occurrence of lymphedema and grading of lymphedema.

Arm circumferences were measured with inch tape at predetermined sites on the side of mastectomy. The sites of measurement were the standard point of reference that was reproducible. The arm was measured circumferentially at 10 cms above and below olecranon process and; at elbow, wrist, interphalangeal thumb and mid palm. More than 2 cms difference in the arm circumference on the side of mastectomy with reference to the unaffected arm was considered as lymphedema. It was a reliable method (r=0.9) to check the arm circumference. The presence and absence was coded as 1 and 2 respectively.

The Lymphedema grading scale which was used for this study was developed by Lee, Morgan and Bergan (1992) and endorsed by the American society of lymphology. It consisted of five items namely grade I, II, III a, III b, and IV. It was graded based on the severity of lymphedema with reference to the healthy arm circumference. The grading of lymphedema was scored with the maximum and minimum of 5 and 1 respectively.

*Part E: Assessment of Quality of life-structured self-administered questionnaire*

The functional assessment of cancer therapy – Breast cancer (FACT -B) version 4 is a systematic collection of health-related quality of life (HRQOL) questionnaires. It was developed in 1987 and modified in 2003 by Kimberly Webster, David cella and Kathleen Yost through FACIT measurement system research programme. It was a compilation of 27 questions, divided into 4 primary quality of life domains; physical, social/ family, Emotional and functional well being. There are 7 items each in physical, social wellbeing and functional wellbeing and 6 items in emotional wellbeing. 

Further it included a subscale of 9 items specifically for cancer breast symptom indices. The breast cancer subscale addressed the questions associated with adverse effects of breast cancer and therapy, such as hair loss, changes in weight, and body image. The FACT-B had 36 items in total. The Reliability was computed by test re -test method by Pearson’s correlation coefficient and r= 0.89.

The FACT-B used the 5-point Likert scale each item had a possible score of 0-4, corresponding to the phrases not at all 0, a little bit 1, somewhat 2, quite a bit 3 and very much 4. Participants choose the number corresponding to how true each statement has been for them during the last 7 days. The scores from the 36 items were given equal weight and then summed to create a total FACT-B score. The total FACT-B score had a range of 0 to 144 with a higher number correlating to a more favorable quality of life. The total score was interpreted as the higher the score better the quality of life. 

*Data collection procedure*

Informed written consent was obtained from the study participants. The investigator conducted pretest for both the study and comparison group in preoperative period 5 days prior to the mastectomy using structured instrument to assess the background variables, arm circumference and quality of life. The data related to demographic variables were collected using interview technique, clinical variables were obtained from clinical records, arm circumference was measured with inch tape, and quality of life through self -administered questionnaire. Post test 1 (02), 2 (03), 3 (04) and 4 (05) were conducted for both study and comparison group at 10^th^, 30^th^, 60^th^ and 90^th^ post operative day respectively.

The descriptive and inferential statistics were used to analyze the data by using SPSS version 23.0.

## Results

Figure no 1 illustrates that none of the study group participants developed lymphedema up to post test 4 because, they were subjected to lymphedema prevention protocol whereas in comparison group 9 (15.0%) developed at post test 4. The Figure number 1 denotes the distribution of lymphedema occurrence among study and comparison group in post test 4.The distribution of grading of lymphedema revealed that 9 (7.5%) subjects in comparison group developed Lymphedema in post test 4 which were in grade I.

The Table 4 depicts that there was a statistically substantial difference between study and comparison group over a period of time on lymphedema occurrence at p < 0.01. It unveils that lymphedema prevention protocol is effective among study group participants. Hence H_1_ –“There is a significant difference in lymphedema occurrence among patients undergone mastectomy and received lymphedema prevention protocol than those who do not at p< 0.05” is accepted.

The independent ‘t’ value on comparison of lymphedema occurrence between study and comparison group in post test 4 showed that there was a statistically significant difference at p< 0.01. The independent ‘t’ value on comparison of pre and post test scores of quality of life between study and comparison group denotes that there was a statistically significant difference in post test 1,2,3 and 4 at p< 0.001. The comparison of physical wellbeing between study and comparison group subjects illustrated statistically significant difference in post test 1, 2, 3 and 4 at p< 0.001. The statistically significant difference was elicited in social wellbeing between study and comparison group subjects in post test1at p< 0.01. 

There was a statistically significant difference in emotional wellbeing between study and comparison group subjects in post test 2, 3 and 4 at p< 0.001. The comparison of functional wellbeing between study and comparison group subjects unveiled statistically significant difference post test 1, 2, 3 and 4at p< 0.001. There was a statistically significant difference in breast cancer subscale between study and comparison group subjects in post test 2 at p< 0.05.

The Table 5 on repeated measures of ANOVA revealed a substantial difference between study and comparison group over a period of time on total score of quality of life, physical wellbeing, emotional wellbeing and functional wellbeing which was statistically significant at p< 0.001. It shows that lymphedema prevention protocol is effective among study group. Thus H_2_- “There is a significant difference in quality of life among patients undergone mastectomy and received lymphedema prevention protocol than those who do not at p<0.05” is accepted.

## Discussion

*The first objective was to identify the effect of lymphedema prevention protocol on lymphedema occurrence among patients undergoing mastectomy*

The results of this study affirmed that the systematic execution of lymphedema preventive measures meticulously at an earlier stage prevented the lymphedema occurrence among study group participants which will be effective throughout their survivorship. Further the univariate analysis demonstrated no significant difference between study and comparison group on age, BMI and axillary lymph node dissection status which are the independent predictor of development of lymphedema. These findings proclaimed that the lymphedema was prevented only because of the execution of lymphedema prevention protocol among study group participants. Many research studies identified the effectiveness of lymphedema prevention protocol prospectively and proved its effectiveness progressively. 

The present study results are substantiated by the another similar study regarding prospective evaluation of lymphedema prevention protocol on lymphedema occurrence at 1, 3, 6, 12 and 24 months post operatively revealed that out of 55 women following surgery for breast cancer the occurrence of lymphedema was 8% in protocol group and 33% in comparison group women. Thus it proved that the proactive lymphedema preventive measures are very effective in preventing lymphedema among patients undergoing mastectomy (Boccardo et al., 2009). 

In addition to the above findings the repeated measures of ANOVA on comparison of lymphedema occurrence among study and comparison group also depicted that there was a statistically substantial difference over a period of time at p< 0.01.These findings disclosed that lymphedema prevention protocol demonstrated the statistically significant difference between study and comparison group on occurrence of lymphedema on long term basis .

Further these study findings were substantiated by the study findings which disclosed that early measures to reduce the risk of lymphedema after surgery for breast cancer significantly reduced the risk (p=0.01, risk ratio 0.28) among intervention group of women than in comparison group. It is evident from these findings that when the lymphedema risk reduction measures are taken pre operatively it yields good outcome in terms of prevention of lymphedema (Lacomba et al., 2010).

A similar study which was conducted to identify the effect of upper extremity exercises on secondary lymphedema in breast cancer patients concluded that there was a statistically significant difference in arm volume at p < 0.01 in study group and showed trends towards increased physical functioning (p = 0.050), general health (p = 0.048) and vitality (p= 0.023) (McKenzie and Kalda, 2003).

*The second objective was to determine the effect of lymphedema prevention protocol on quality of life among patients undergoing mastectomy.*

When independent ‘t’ test was used to compare the pre and post test scores of quality of life (QOL) between study and comparison group, it denoted that there was a statistically significant difference between study and comparison group participants, in post test 1, 2, 3 and 4 at p< 0.001. It was noticeable from these findings that the QOL was better among study than in comparison group. These study findings are consistent with the study conducted to analyze the effectiveness of decongestive therapy in the prevention of lymphedema secondary to mastectomy which revealed that after the intervention period the study group showed significant difference (p< 0.05) in the quality of life and functional assessment of the volume of the limb of the mastectomy side (Castro-Sánchez et al., 2011).

Another study conducted to examine the effect of a home based exercise programme on lymphedema and quality of life in post mastectomy patients revealed that there was a statistically significant improvement in the affected upper-limb circumference and volume (p< 0.001) and in the quality of life scores (p< 0.001) (Gautam et al., 2011).

When the repeated measures of ANOVA was used it disclosed that there was a substantial difference between study and comparison group over a period of time on quality of life subscales; physical wellbeing, emotional wellbeing and functional wellbeing, which were statistically significant at p< 0.001. Further these findings are supported by a study conducted on quality of life among breast cancer patients with lymphedema; a systematic review of patient reported outcome instruments and outcomes. The results revealed that 16 of 39 studies were complaint with the efficacy criteria. Exercise and Complex Decongestive Therapy interventions were associated with improved HRQOL (Pusic et al., 2013). These findings are substantiated by a study conducted to evaluate the perioperative training for lymphedema assessment and protection. The incidence of lymph edema was 58.3% with a majority occurring within the first year after surgery (78.4%). Majority of the patients developed acute lymphedema by three months and QOL was worst in comparison group than in study group (Becker et al., 2006).

Further these findings were supported by a study conducted to identify the effect of strength training exercises on health related quality of life in breast cancer related lymphedema patients. The randomized controlled trial study was conducted with the total of 234 participants after the breast cancer treatment of 78 months. The findings revealed that the health related quality of life scores improved significantly with twice weekly strength training exercises regardless of lymphedema status. The health related quality of life domains such as social, appearance, sexuality, psychosocial, physical and body image were improved which was statistically significantly at p<0.05 (Speck et al., 2010). 

It is validated from the above that the lymphedema prevention protocol was effective to promote the quality of life among breast cancer patients who are undergoing mastectomy. Hence, “H_2_ - There is a significant difference in quality of life among patients undergone mastectomy and received lymphedema prevention protocol than those who do not at p<0.05” is accepted.

*“Prevention is better than cure”*

Lymphedema is a chronic debilitating problem which can never be cured and adversely affects their quality of life. Every patient is entitled to have information about their own care during the course of treatment and each nurse is accountable for the same. These study findings proclaimed that early implementation of lymphedema prevention protocol was very effective in terms of prevention of lymphedema and enhancement of QOL among patients undergone mastectomy which will yield the favorable clinical outcome throughout their survivorship. 
